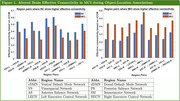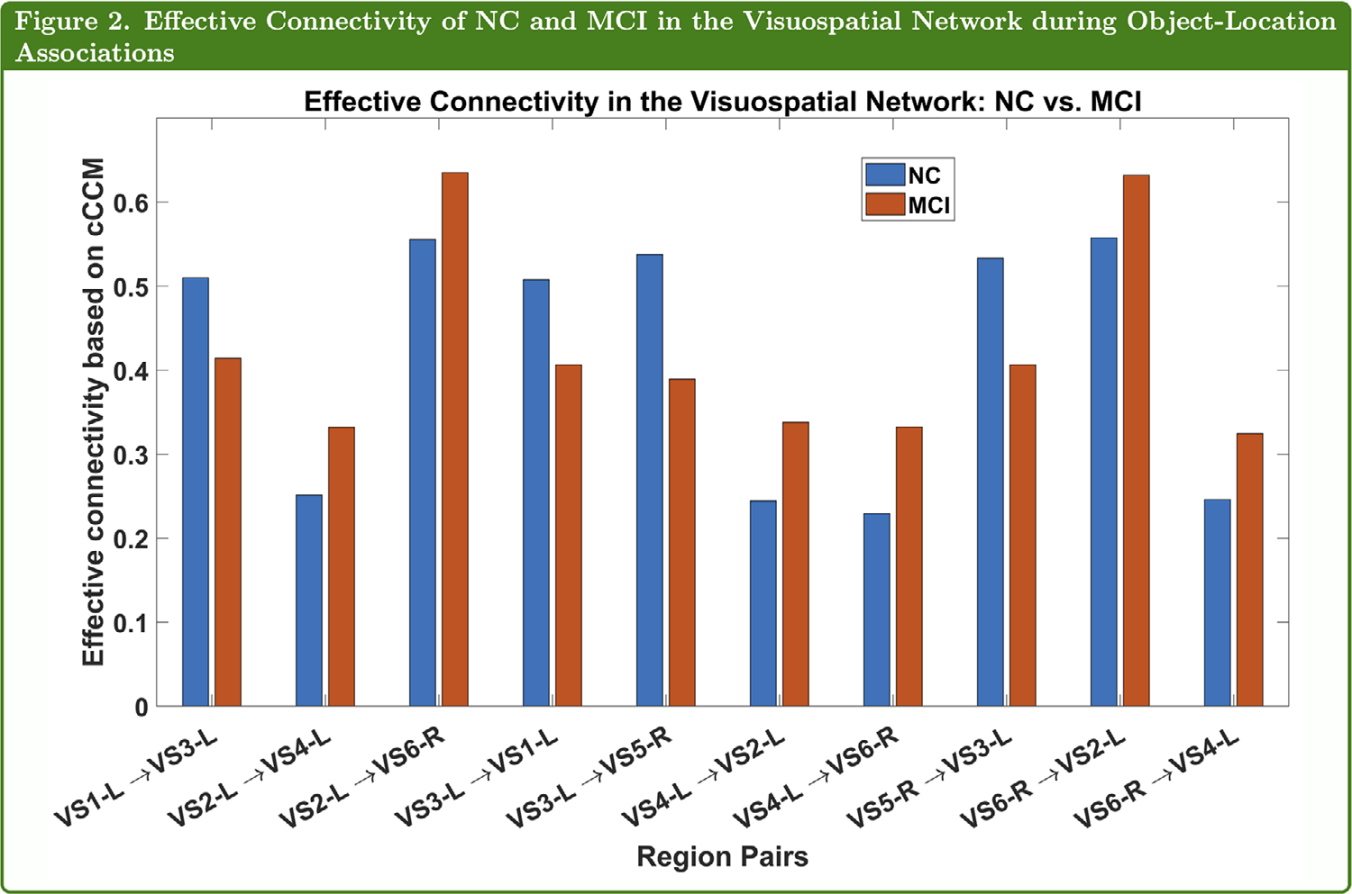# Altered Brain Effective Connectivity in MCI during Object‐Location Associations: an fMRI Study

**DOI:** 10.1002/alz.095799

**Published:** 2025-01-09

**Authors:** Boxin Sun, Jinxian Deng, Norman Scheel, Arijit K Bhaumik, Jian Ren, Benjamin M. Hampstead, Tongtong Li

**Affiliations:** ^1^ Michigan State University, East Lansing, MI USA; ^2^ University of Michigan Medical School, Ann Arbor, MI USA; ^3^ Michigan Alzheimer’s Disease Research Center, Ann Arbor, MI USA; ^4^ University of Michigan, Ann Arbor, MI USA

## Abstract

**Background:**

Existing work suggests that Alzheimer’s disease (AD) pathology can affect the direction and intensity of information signaling in functional brain regions. This study aims to explore how mild cognitive impairment (MCI) can affect the brain effective connectivity.

**Method:**

We used an event‐related functional magnetic resonance imaging (fMRI) paradigm to compare patients with aMCI and healthy controls with normal cognition (NC) as they encoded 90 ecologically‐relevant object‐location associations (OLAs). Two additional OLAs, repeated a total of 45 times, served as control stimuli. Memory for these OLAs was assessed following a 1‐hour delay. The groups were well matched on demographics and brain volumetrics. A total of 44 right‐handed participants (19 NC, 25 aMCI, age mean: 71.5) completed this study.

We evaluated the effective connectivity between all the possible functional brain region pairs using causalized convergent cross mapping (cCCM), which measures the intensity of directional information transfer between the brain regions.

**Result:**

The region pairs where the effective connectivity (in terms of cCCM values) of NC and MCI exhibit the largest, statistically significant differences (p‐value < 0.05) are shown in Figure 1, and the result for the visuospatial network alone is shown in Figure 2. Our analysis shows that during the encoding of ecologically relevant object‐location associations, MCI demonstrated statistically significant reduction in effective connectivity (p‐value < 0.05) across some regions, but also showed increased effective connectivity across other regions.

**Conclusion:**

The increased effective connectivity of MCI in some regions indicates the presence of detours in information transfer due to the weakening of connectivity in other regions and may reflect the compensatory mechanism of the brain.